# Real-World Efficacy and Safety of Fluocinolone Acetonide Implant for Diabetic Macular Edema: A Systematic Review

**DOI:** 10.3390/pharmaceutics13010072

**Published:** 2021-01-07

**Authors:** Laurent Kodjikian, Stephanie Baillif, Catherine Creuzot-Garcher, Marie-Noëlle Delyfer, Frédéric Matonti, Michel Weber, Thibaud Mathis

**Affiliations:** 1 Department of Ophthalmology, Croix-Rousse University Hospital, Hospices Civils de Lyon, University of Lyon 1, 69004 Lyon, France; mathisthibaud@hotmail.fr; 2 CNRS-UMR 5510 Mateis, University of Lyon 1, 69100 Villeurbane, France; 3 Department of Ophthalmology, Pasteur 2 University Hospital, 06000 Nice, France; baillif.s@chu-nice.fr; 4 Department of Ophthalmology, Dijon-Bourgogne University Hospital, 21000 Dijon, France; catherine.creuzot-garcher@chu-dijon.fr; 5 Eye and Nutrition Research Group, CSGA, UMR1324 INRA, 6265 CNRS, Burgundy, 21000 Dijon, France; 6 Department of Ophthalmology, Bordeaux 2 University Hospital, 33000 Bordeaux, France; marie-noelle.delyfer@chu-bordeaux.fr; 7 Bordeaux Population Health Research Center, Team LEHA, 33000 Bordeaux, France; 8 Monticelli Paradis Center, 13000 Marseille, France; frederic.matonti@free.fr; 9 Institut de Neurosciences de la Timone-UMR 7289, University of Aix-Marseille, 13000 Marseille, France; 10 Department of Ophthalmology, Nantes University Hospital, 44000 Nantes, France; michel.weber@chu-nantes.fr; 11 Clinical Investigation Centre CIC1413, INSERM and Nantes University Hospital, 44000 Nantes, France

**Keywords:** fluocinolone acetonide, FAc, efficacy, safety, real-world, diabetic macular edema, DME

## Abstract

To assess real-world outcomes of fluocinolone acetonide (FAc) implant in treating diabetic macular edema (DME), a systematic literature review was conducted on PubMed in order to identify publications assessing the efficacy and safety of the FAc implant in DME in daily practice. Case reports and randomized controlled trials were excluded. Twenty-two observational real-world studies analyzing a total of 1880 eyes were included. Mean peak visual gain was +8.7 letters (11.3 months post-FAc injection) and was greater for lower baseline best corrected visual acuity (BCVA) and for more recent DME. Mean central retinal thickness (CRT) decreased 34.3% from baseline. 77.0% of the analyzed studies reported both BCVA improvement of at least five letters and a CRT decrease by 20% or more. Rescue therapy was needed more frequently when FAc was administered for chronic DME. FAc-induced ocular hypertension was reported in 20.1% of patients but only 0.6% needed surgery. Cataract extraction was performed in 43.2% of phakic patients. Adequate patient selection is essential for optimal FAc response and better safety profile. Currently positioned as second- or third-line treatment in the management algorithm, FAc implant decreases treatment burden and provides better letter gain when administered for more recent DME.

## 1. Introduction

According to the International Diabetes Federation, 700 million adults are expected to be living with diabetes by 2045 [1]. Diabetic macular edema (DME) directly impairs central vision making diabetes the leading cause of severe visual impairment in working-age populations of developing countries [2,3]. Although its pathogenesis is multifactorial, it mainly results from vasogenic changes secondary to hyperglycemia and to increased levels of vascular endothelial growth factor (VEGF-A). It results in increased retinal hypoxia that induces a breakdown in the blood retinal barrier and an accumulation of fluid in the macular region [3,4,5,6,7]. Over the last decade, anti-VEGF injections such as ranibizumab, aflibercept, or bevacizumab targeted this growth factor and proved to be more efficient and safer than the traditional focal/grid laser photocoagulation [3,5,8,9,10,11].

However, studies showed that around 30% of DME patients are non-responders to repeated intravitreal anti-VEGF injections [6,12,13]. This can be explained by the persisting low-grade inflammatory process that was identified in the pathogenesis of DME. Corticosteroids provide an alternative therapeutic strategy, particularly in chronic DME and in patients resistant to anti-VEGF treatment. Not only do they suppress the inflammatory process by inhibiting multiple chemokines and inflammatory cytokines, but they also interfere with other pro-inflammatory molecules such as VEGF-A, reducing vascular permeability and suppressing angiogenesis [14,15,16,17,18].

Two slow release sustained delivery systems of intraocular steroids were developed. The 0.7 mg intravitreal dexamethasone (DEX) implant (Ozurdex^®^, Allergan Inc., Irvine, CA, USA) enables extended drug release over a 6-month period [16,19,20,21]. The 0.19 mg intravitreal fluocinolone acetonide (FAc) implant (Iluvien^®^, Alimera Sciences Ltd., Alpharetta, GA, USA) provides an average release rate of 0.2 μg per day for the first 3 months followed by a maintained concentration of 0.5–1.0 ng/mL for up to 36 months [15,16,17,22,23,24].

Strict patient selection and close follow-ups used in the FAc FAME A and B randomized controlled trials (RCTs) make it hard to extrapolate their findings to real-life routine practice where patients might have co-morbidities such as ocular hypertension (OHT), poor diabetes and blood pressure conditions with increased risk of loss of follow-up. Therefore, findings from “real-life” observational studies, even though of a lower level of evidence compared to interventional trials, are useful to complete our understanding of patients in routine practice. An analysis of a significant number of these real-life studies is necessary to minimize biases such as loss of follow-up and missing data and to draw valid conclusions.

The objective of this work is therefore to combine data from available real-world observational studies concerning the FAc implant in order to draw potential trends and conclusions concerning its efficacy and safety in treating DME in daily practice.

## 2. Materials and Methods

A search of PubMed was performed in March 2020 using the keywords (“Fluocinolone Acetonide” OR “Iluvien”) AND (“DME” OR “DMO” OR “Diabetic Macular Edema”) in order to identify publications assessing the efficacy and safety of the FAc implant on DME and retrieved 64 results. Only publications that were not solely evaluating the economic impact of the implant, which were also not case report studies, RCTs or subgroup analysis of RCTs, were selected. Of these, only articles published in English, those with a follow-up period of more than six months and a global population of more than 10 eyes with DME were selected. A total of 22 studies met all inclusion criteria and were included in the final analysis. When different patient subgroups were found for any given study, results were presented separately for each subgroup. Because of the nature of this work, no ethics committee approval was obtained.

To report on functional and anatomical efficacy, primary analysis was performed on the FDA-validated criteria used in the Reinforce study for corticosteroid slow release sustained delivery systems, using the maximum functional efficacy (peak) regardless of the population of patients followed at this endpoint [25]. For analysis purposes, best corrected visual acuity (BCVA) was converted from logMAR to the Early Treatment Diabetic Retinopathy Study (ETDRS) score when necessary. Similarly, the mean maximum improvement of central retinal thickness (CRT) was reported. Secondary analysis was performed on subgroups according to baseline BCVA (less than 50 letters, between 50 and 60 letters and greater than 60 letters) and to DME duration (less than 2 years, between 2 and 4 years and greater than 4 years). The mean percentage of patients who needed additional treatments during the follow-up period was also analyzed.

To report on safety, especially concerning intraocular pressure (IOP) and lens status, the following variables and demographic characteristics were analyzed: mean percentage of patients with OHT at inclusion; mean percentage of FAc-induced OHT as defined by each paper’s criteria; mean percentage of patients who needed anti-glaucoma medications and/or glaucoma surgeries; mean percentage of phakic patients at inclusion; mean percentage of lens opacification and/or cataract surgery; and rate of endophthalmitis following the intravitreal injection.

## 3. Results

This section may be divided by subheadings. It should provide a concise and precise description of the experimental results, their interpretation as well as the experimental conclusions that can be drawn.

### 3.1. Study Population

All 22 observational real-world studies retained used FAc implants as second- or third-line treatment for DME, after anti-VEGF injections and/or steroid treatment (DEX implants and/or triamcinolone injections). A total of 1880 eyes from 1675 DME patients were included in the final analysis (Table 1).

#### 3.1.1. Efficacy

##### Visual Acuity

**Figure 1:**
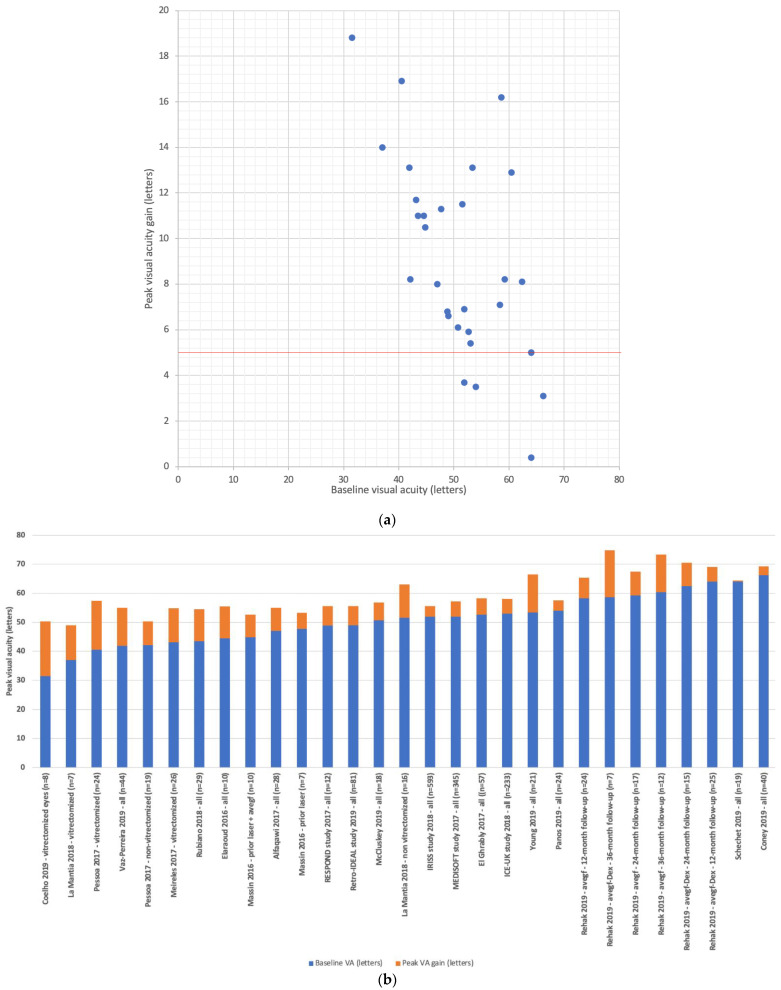
Summary of real-world studies evaluating the functional efficacy of the fluocinolone acetonide implant in the treatment of diabetic macular edema. (**a**) peak visual acuity; (**b**) peak visual acuity gain. Mean follow-up was 20.0 months (*n* = 22 studies).

**Figure 2:**
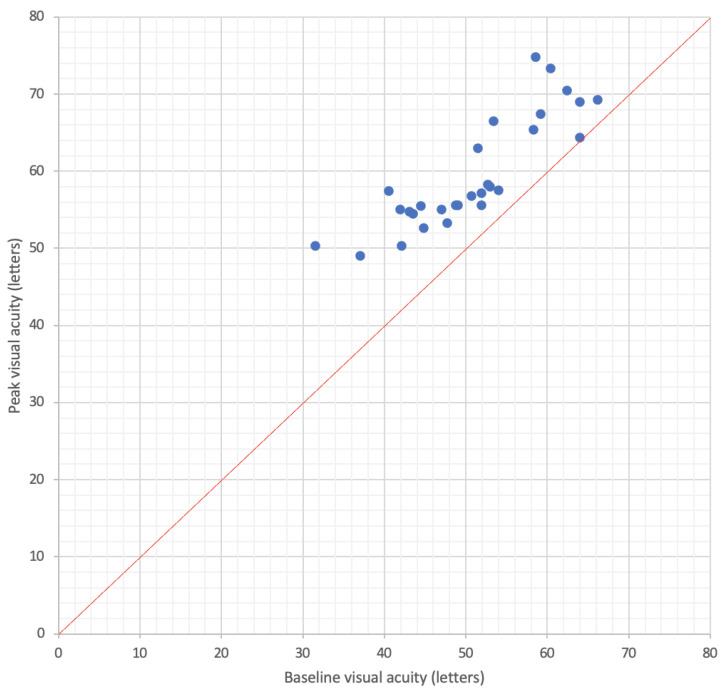
Peak visual acuity as a function of baseline visual acuity in real-world studies evaluating the efficacy of the fluocinolone acetonide implant for diabetic macular edema. Mean follow-up was 20.0 months (*n* = 22 studies).

Studies that evaluated the functional efficacy of the FAc implant (*n* = 22 studies) had a mean follow-up of 20.0 months (range: 8.5–36.0 months, median: 18.0 months) [15,26,27,28,29,30,31,32,33,34,35,36,37,38,39,40,41,42,43,44,45]. Mean baseline BCVA was 50.8 letters (range: 45.7–71.0 letters, median: 51.6 letters) and improved to a maximum of 59.5 letters (range: 49.0–74.8 letters, median: 57.2 letters) after FAc implant injection. All except four studies reported a BCVA improvement of five letters or more after FAc implant injection (Figure 1). The mean peak visual gain of +8.7 letters (range: 0.4–18.8 letters, median +8.0 letters) was observed at 11.3 months (range: 3.0–36.0 months, median: 9.0 months) (Figure 2).

Subgroup analysis of the peak BCVA gain according to the baseline BCVA and DME duration is summarized in Table 2a. Greater BCVA gain was observed for lower baseline BCVA (+11.0 letters for a baseline BCVA <50 letters and +7.0 letters for a baseline BCVA <60 letters) and for more recent DME (+8.7 letters and +9.4 letters for a DME duration between 0 and 2 years and between 2 and 4 years respectively, and +5.1 letters for a DME duration >4 years).

##### Retinal Thickness

**Figure 3:**
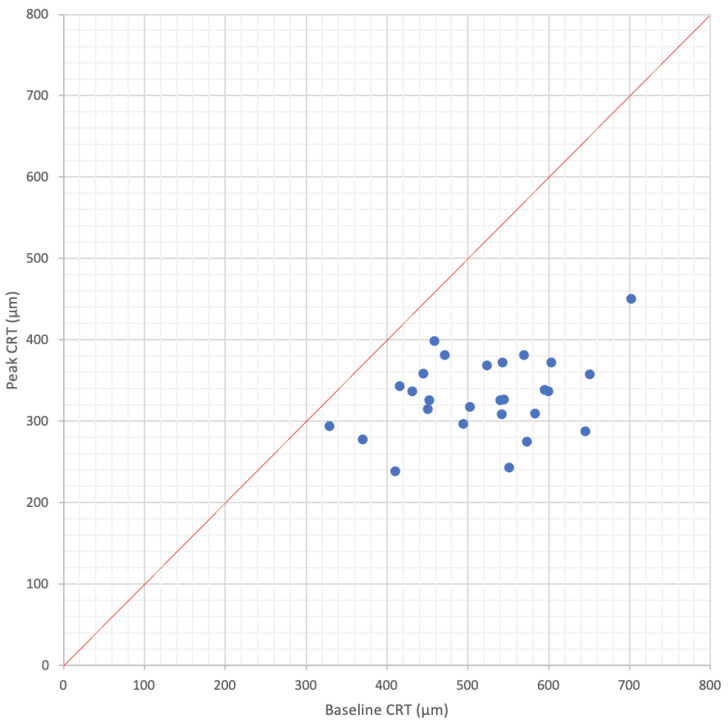
Peak central retinal thickness as a function of baseline central retinal thickness in real-world studies evaluating the efficacy of the fluocinolone acetonide implant for diabetic macular edema. Mean follow-up was 20.3 months (*n* = 20 studies).

Studies that evaluated the anatomical efficacy of the FAc implant (*n* = 20 studies) had a mean follow-up of 20.3 months (range: 8.5–36 months, median: 21.0 months) [26,27,28,29,30,32,33,34,35,36,37,38,39,40,41,42,43,44,45,46]. Mean baseline CRT was 516 µm (range: 328–701 µm, median: 532 µm) and decreased to a minimum of 332 µm (range: 237–450 µm, median: 331 µm) after FAc implant injection (Figure 3). Maximum decrease of −34.3% from baseline (range: −10.7–−55.8%, median: −36.2%) was observed at 16.6 months (range: 4.0–36.0, median: 12.0 months). A subgroup analysis of the peak CRT decrease according to baseline CRT is summarized in Table 2b. Greater percentage of CRT decrease was observed for thicker baseline CRT (−44%) in comparison with thinner baseline CRT (−18%).

##### Visual and Anatomical Correlation

**Figure 4:**
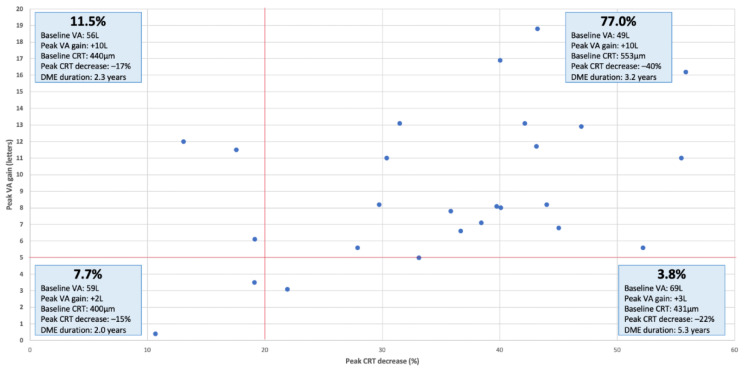
Peak visual acuity gain as a function of peak central retinal thickness decrease in real-world studies evaluating the efficacy of the fluocinolone acetonide implant for diabetic macular edema. Mean follow-up was 20.2 months (*n* = 18 studies). Top left, functional responders; top right, anatomical and functional responders; bottom left, anatomical and functional non-responders; bottom right, anatomical responders.

Of the 22 studies retained, only those assessing both functional and anatomical efficacy were used for this analysis (*n* = 18 studies) [26,27,28,29,30,32,33,34,35,36,37,38,39,40,41,42,43,44,45]. Functional efficacy was defined as a BCVA improvement of at least five letters, while anatomical efficacy as a CRT decrease by 20% or more (Figure 4) similarly to the definitions used by the DRCRnet study group [47,48]. Mean follow-up duration for these studies was 20.0 months (range: 8.5–36.0 months, median: 18.0 months). An anatomical-functional correlation was seen in 77.0% of the studies with a mean CRT decrease of −40.5% from baseline (range: −27.9–−55.8%, median: −40.0%) and a mean BCVA gain of +10.1 letters (range: +5.0–+18.5 letters, median: +8.2 letters).

##### Additional Treatments

**Figure 5:**
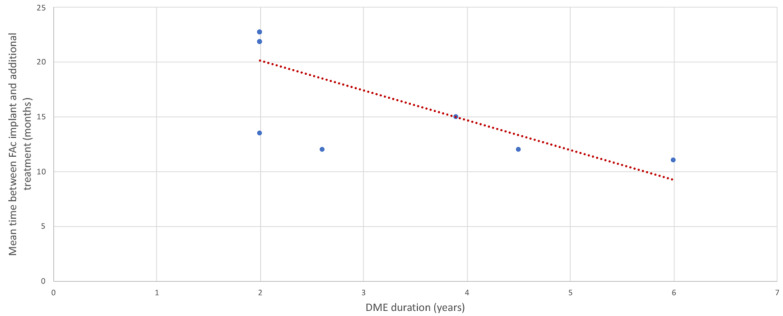
Mean time between fluocinolone acetonide implant and initiation of additional intravitreal DME treatments according to chronicity of diabetic macular edema at the time of injection, in real-world studies evaluating the efficacy of the implant for diabetic macular edema (*n* = 6 studies).

Almost 30.0% of patients (range: 7.0–54.2%, median: 30.9%) needed one or more additional intravitreal DME treatment during the follow-up period. The average duration between treatments post-FAc implant injection was 15.4 months (range: 11.0–22.7 months, median: 13.5 months) and was inversely proportional to the DME duration (Figure 5).

##### Safety

Intraocular Pressure

Almost one out of four injected patients (Mean 25.8%) had OHT at inclusion (range: 0.0–83.3%, median 22.2%). A total of 20.1% of patients (range: 6.9–47.4%, median: 17.5%) had a FAc-induced OHT during the follow-up period. IOP-lowering medication was needed in 23.4% (range: 0.0–62.5%, median: 23.8%) and only 0.6% of patients needed IOP-lowering surgery (range: 0.0–4.7%, median: 0.0%).

Cataract

Concerning steroids-induced cataracts, 18.8% of patients included were phakic (range: 0.0–54.2%, median: 16.4%) at baseline. Of those, lens opacification was observed in 31.47% of cases (range: 0.0–100.0%, median: 21.3%) and 43.2% (range: 0.0–100.0%, median: 33.9%) needed cataract surgery. The mean time between FAc implant injection and cataract surgery was 8.1 months (range: 5.25–13.4 months, median: 6.8 months) [34,39,42,44].

Endophthalmitis

Infectious complications were not described in all studies. Only one patient with poorly controlled diabetes developed an endophthalmitis post injection and was well managed with antibiotic treatment [26].

## 4. Discussion

Anti-VEGF and DEX implants are both approved as first line treatments for center-involving DME but require regular repeated injections to maintain sufficient concentrations in the posterior segment of the eye [49]. The FAc implant demonstrated anatomical and functional efficacy in DME patients for up to 3 years of follow-up and is now indicated as second- or third-line treatment in the management algorithm [23]. The rationale for using corticosteroids in the treatment of DME is on the one hand its powerful anti-inflammatory and anti-edematous effects. Corticosteroids suppress the inflammatory process of DME by inhibiting prostaglandins, proinflammatory and angiogenetic mediators such as IL-6, IL-8, MCP-1, ICAM-1, TNF-α and VEGF-A in both in vitro and in vivo settings. They also change the local ratio of laminin isoforms in the endothelial basal membrane, improving the blood retinal barrier and limiting permeability and leakage by strengthening capillaries tight junctions. They finally inhibit the inflammatory processes caused by activated Müller glial cells that may become apoptotic with disease progression and alter the homeostasis of the retina [50,51,52,53]. On the other hand, anti-VEGF demonstrated poor results in real-life studies with a mean number of six letters gain due to a reduced number of injections; the discordance between RCTs and real-life outcomes, a major issue with anti-VEGF treatments, being mainly due to poor compliance with the tight schedule of monitoring and injections that is mandatory to achieve best outcomes [13].

Because of the high lipophilic nature of FAc compared to dexamethasone, adequate penetration and accumulation in the retina is possible after continuous low doses release [19,54]. This allows for fewer injections needed, less injection-related complications and a reduction of treatment frequency by up to 87% [43,46]. Considering that 42% of a European sample of 131 retinal patients would prefer having fewer injections for the same results, one can see FAc implants as a way to decrease treatment burden and improve patient’s quality of life [55]. However, our work shows that intravitreal FAc injection is also effective in improving BCVA and reducing CRT, highlighting its particular interest in “real-world” DME patients.

In fact, all analyzed studies reported an improvement of the BCVA with a gain that ranged from +0.4 letters to +18.8 letters at peak efficacy. The mean peak gain of +8.7 letters observed in the “real-world” setting is higher than the +5.3 letters gain reported by Campochiaro et al. in their pivotal FAME study, despite similar baseline visual acuity [23]. This discrepancy can be explained by the strict and specific inclusion criteria of therapeutic trials that do not necessarily represent all patients of routine practice, and by the possibility to reinject more often and as needed in real-world conditions. Furthermore, this peak visual acuity gain is similar to the one reported for DEX implants (+9.6 letters) and twice as high as the one with anti-VEGF (+4.7 letters) [13]. FAc implants are used as second- or third-line treatment in the management algorithm and are therefore injected for relatively “chronic” DME. Our results show that not only do they decrease treatment burden, but they also allow for visual improvement similar to first- or -second-line treatments used earlier in the course of the disease.

In our review, the gain was greater for lower baseline BCVA, but also for more recent DME. Indeed, FAc implant injections for DME older than four years were still successful with a +5.1 letters gain, but results were less impressive than for more recent DME. Recurrent DME causes architectural changes in the retina with ganglion cell-inner plexiform layer thinning, glial proliferation, and photoreceptor damage at the level of the fovea that are correlated with poor visual recuperation despite a favorable anatomical response [56,57,58,59,60]. A more recent study also showed ganglion cell layer and retinal nerve fiber layer thinning in eyes of diabetic patients with faster retinal neurodegeneration once diabetic retinopathy (DR) develops [61].

On an anatomical level, FAc implant led to a 34.7% decrease of CRT. These results were concordant with Campochiaro et al. who reported a 37.9% decrease from baseline [23]. In their study, Schechet et al. reported a significant decrease of mean CRT and less CRT amplitude fluctuation after FAc over a mean follow-up period of 399 days [43]. FAc implant provides long-term stabilization of CRT for up to 3 years, which could theoretically limit the structural damages at the level of the fovea and allow for better visual recovery.

To our knowledge, this is the first review that demonstrates a potential functional and anatomical response correlation of FAc implant despite its positioning in the management algorithm of DME. This correlation was present for almost three quarters of the included studies (77.8%) with both a BCVA gain of ≥5 letters and a CRT decrease of ≥20%. In other terms, 8 out of 10 eyes with chronic or refractory DME had a favorable anatomical and functional response to FAc. Overall, better functional response was observed in studies with worse baseline BCVA. Due to the architectural damage in the retina described above, patients with a longer DME duration (more than 5 years) had anatomical improvement but did not gain much visual acuity. Patients with higher baseline BCVA had fewer letters gain but still achieved similar peak results. These “real-world” results were also described by Eaton et al. in their USER study, they reported improvement of BCVA for patients with the poorest VA at the time of FAc implant administration and stabilization of BCVA for patients with better baseline VA [46].

Intravitreal additional treatments after FAc injection were needed for almost a third of the patients. Eaton et al. demonstrated that FAc implant injection led to a significant decrease of treatment frequency (from 2.9 months to 14.3 months post-FAc implant injection) and consequently to less treatment burden [46]. Furthermore, our review showed that FAc yielded better visual improvement and was sufficient as a monotherapy for a longer period of time when administered for more recent DME. Therefore, it would be reasonable to switch to FAc injections earlier in the treatment regimen when DME is shorter in duration in order to delay the need for rescue therapy.

OHT is a major safety concern of steroids intravitreal injections. DEX sustained slow release systems should be avoided in patients with advanced or uncontrolled glaucoma or under at least dual therapy [49]. Studies have shown that patients with prior steroid treatment who did not developed drug-induced OHT are at low risk of developing it following the FAc implant [32,46]. Despite this high positive predictability, the latter is contraindicated in glaucomatous patients. Interestingly though, almost one out of four “real-world” patients (25.8%) receiving the implant had an elevated IOP >21 mmHg at baseline, which was an exclusion criterion in the pivotal FAME study. It highlights even more the importance of observational studies in mirroring real-life routine practice. Only 20.1% of DME patients experienced FAc-induced OHT and 23.4% needed IOP-lowering drugs. Despite having patients with higher baseline IOP, our review shows a safer pressure profile when compared to Campochiaro et al.’s pivotal study. This is particularly obvious in the percentage of patients who needed IOP-lowering surgeries (0.6% in our review versus 4.8% in FAME) [23]. Therefore, in addition to maintaining a systematic quarterly follow-up for IOP monitoring, careful patient selection based on prior steroid-induced OHT seems essential. Otherwise, assessment by optical coherence tomography of retinal nerve fiber layer thickness could be of interest to evaluate early glaucomatous alteration. Depending on local guidelines this evaluation should be done for each follow-up visit in case of IOP increase.

Cataract extraction was needed in 43.2% of phakic patients, which is much lower than the rate reported in the FAME study (80.0%). In their retrospective chart review, Rehak et al. highlighted the impact of cataract progression on BCVA with restoration of vision to levels significantly higher than pre-FAc administration after phacoemulsification [40]. Pseudophakic patients, on the other hand, presented long-term stable visual and anatomical improvement. These findings are also consistent with the FAME study that reported a drop in BCVA at 9–18 months due to lens opacification, followed by an improvement after cataract surgery between 18 and 24 months [23].

Lastly, concerning infectious complications and the FAc implant safety profile, only one case of endophthalmitis has been reported, which did not allow for any statistical analysis.

Our review has several limitations. “Real-world” observational studies are important in completing pivotal studies by including real-life patients, but their statistical analysis can be biased by missing data and patient’s loss of follow-up. Furthermore, studies included were heterogenous with different primary endpoints (anatomical, functional or both). Some pieces of information were missing and could not be assessed for all studies such as duration of DME or time before initiation of additional treatment. Moreover, DME is known to be affected by hypertension, hyperlipidemia, and renal function but real-world studies only inconsistently report these data. We could also have reported the evolution of the macular volume on SD-OCT, more precise than retinal thickness. It has already been shown a correlation between FAc injection and the decrease of this parameter; however, too few studies have analyzed this parameter [35,41,44]. Another limitation is the relatively small sample of eyes included in some studies which limits the generalizability of the results. Nevertheless, many of these biases were compensated by the fact that we analyzed a significant number of studies gathering almost 2000 eyes.

## 5. Conclusions

In conclusion, the real-life outcomes of FAc injections are comparable if not superior to those of interventional trials. Currently positioned as second- or third-line treatment in the management algorithm of DME, FAc implants decrease the treatment burden and still allow for functional and anatomical improvement in such chronic patients, with even better results for DME of shorter duration. Finally, the safety profile seems better than initially thought in RCTs which could be reassuring for physicians.

## Figures and Tables

**Table 1:** Summary of real-world fluocinolone acetonide implant studies.

Study Group	Study Design	N * (Eyes)	Follow-Up (Months)	DME ^†^ Duration (Years)	BL ^‡^ VA ** (Letters)	Final VA (Letters)	Final VA Gain (Letters)	Peak VA (Letters)	Peak VA Gain (Letters)	BL CRT ^††^ (µm)	Final CRT (µm)	Final CRT Decrease (%)	Peak CRT (µm)	Peak CRT Decrease (%)	OHT at BL (%) ^‡‡^	OHT (%)	OHT Meds (%)	OHT Surgery (%)	Phakic at BL (%)	Cataract (%)	Cataract Surgery (%)	Cases of Endopht (n)	Addition. ttt ^†††^ (%)	Time Initiation Addition ttt (Months)
Elaraoud et al. 2016 (all)	Obs ^‡‡‡^-retro ****	10	12	NA ^††††^	44.5	55.0	10.5	55.5	11.0	645.3	287.4	−55.5	287.4	−55.5	40.0	NA	NA	NA	NA	NA	NA	NA	NA	NA
Massin et al. 2016 (prior laser + avegf ^‡‡‡‡^)	Prosp *****	10	12	3.6	44.8	45.7	0.9	52.6	7.8	701.0	450.0	−35.8	450.0	−35.8	0.0	20.0	20.0	0.0	50.0	NA	20.0	NA	30.0	NA
Massin et al. 2016 (prior laser)	Prosp	7	12	7.6	47.7	53.3	5.6	53.3	5.6	573.0	274.0	−52.2	274.0	−52.2	0.0	14.3	14.3	0.0	0.0	NA	NA	NA	14.3	NA
Alfaqawi et al. 2017 (all)	Obs-retro	28	12	6.0	47.0	55.0	8.0	55.0	8.0	494.0	296.0	−40.1	296.0	−40.1	25.0	11.0	11.0	0.0	0.0	0.0	0.0	1.0	7.0	11.0
El Ghrably et al. 2017 (all)	Obs-cons	57	14	2.6	52.7	57.8	5.1	58.3	5.6	452.0	326.0	−27.9	326.0	−27.9	NA	NA	12.3	0.0	22.8	NA	NA	0.0	NA	NA
MEDISOFT study. 2017 (all)	Obs-retro	345	14	NA	51.9	57.2	5.3	57.2	5.3	451.2	355.5	−21.2	355.5	−21.2	14.2	15.4	13.9	0.3	10.4	NA	NA	NA	35.7	. NA
Meireles et al. 2017 (vitrectomized)	Obs-retro	26	8.5	3.7	43.1	54.8	11.7	54.8	11.7	542.0	308.4	−43.1	308.4	−43.1	NA	NA	30.8	0.0	4.0	NA	NA	NA	11.5	NA
Pessoa et al. 2017 (vitrectomized)	Obs-retro	24	24	2.5	40.5	57.4	16.9	57.4	16.9	543.9	326.3	−40.0	326.3	−40.0	37.5	4.2	29.2	0.0	4.2	NA	100.0	NA	8.3	NA
Pessoa et al. 2017 (non-vitrectomized)	Obs-retro	19	24	3.5	42.1	50.3	8.2	50.3	8.2	523.6	368.0	−29.7	368.0	−29.7	31.6	10.5	52.6	0.0	36.8	NA	42.8	NA	26.3	NA
RESPOND study. 2017 (all)	Prosp	12	12	3.4	48.8	52.5	3.7	55.6	6.8	650.5	357.7	−45.0	357.7	−45.0	NA	16.7	NA	0.0	33.0	NA	25.0	NA	NA	NA
ICE-UK study. 2018 (all)	Obs-retro	233	12	2.7	53.0	55.0	2.0	58.0	5.0	NA	NA	NA	NA	NA	19.0	25.0	15.0	0.8	11.0	NA	73.1	NA	30.0	NA
IRISS study. 2018 (all)	Obs-retro	593	24	4.5	51.9	54.8	2.9	55.6	3.7	NA	NA	NA	NA	NA	NA	19.1	23.3	0.8	16.4	NA	NA	NA	31.0	12.0
La Mantia et al. 2018 (vitrectomized)	Obs-retro	7	12	NA	37.0	51.0	14.0	49.0	12.0	459.0	399.0	−13.1	399.0	−13.1	83.3	NA	0.0	NA	0.0	0.0	0.0	NA	NA	NA
La Mantia et al. 2018 (non vitrectomized)	Obs-retro	16	12	NA	51.5	59.5	8.0	63.0	11.5	416.0	344.0	−17.3	343.0	−17.5	6.3	NA	18.8	NA	0.0	0.0	0.0	NA	NA	NA
Rubiano et al. 2018 (all)	Obs-retro	29	36	2.6	43.5	54.5	11.0	54.5	11.0	451.0	314.0	−30.4	314.0	−30.4	6.9	6.9	6.9	0.0	3.0	NA	NA	NA	NA	12.0
USER study. 2018 (all)	Obs-retro	160	24	NA	NA	NA	NA	NA	NA	370.4	276.6	−25.3	276.6	−25.3	NA	35.0	24.4	1.3	22.5	NA	NA	NA	37.0	14.3
Coelho et al. 2019 (vitrectomized)	Obs-retro	8	24	3.9	31.5	49.5	18.0	50.3	18.8	594.8	337.8	−43.2	337.8	−43.2	51.7	12.5	62.5	0.0	6.9	NA	NA	NA	25.0	15.0
Coney et al. 2019 (all)	Obs-retro	40	12	5.3	66.2	67.7	1.5	69.3	3.1	430.9	336.5	−21.9	336.5	−21.9	NA	17.5	12.5	0.0	22.5	0.0	0.0	NA	40.0	NA
McCluskey et al. 2019 (all)	Obs-retro	18	18	2.3	50.7	56.8	6.1	56.8	6.1	444.0	359.0	−19.1	359.0	−19.1	27.8	16.7	27.8	0.0	16.7	NA	NA	NA	44.4	NA
Vaz-Perreira et al. 2019 (all)	Obs-retro	44	24	3.3	41.9	50.2	8.3	55.0	13.1	542.8	421.4	−22.4	372.0	−31.5	18.2	25.0	25.0	2.3	31.8	NA	42.9	NA	NA	NA
Young et al. 2019 (all)	Obs-retro	21	36	2.7	53.4	62.7	9.3	66.5	13.1	410.3	252.5	−38.5	237.5	−42.1	33.0	19.0	38.1	4.7	4.8	NA	NA	NA	23.8	NA

* Number; ^†^ Diabetic macular edema; ^‡^ Baseline; ** Visual acuity; ^††^ Central retinal thickness; ^‡‡^ Ocular hypertension; ^†††^ Treatment; ^‡‡‡^ Observational; **** Retrospective; ^††††^ Not available; ^‡‡‡‡^ Anti-vascular endothelial growth factor; ***** Prospective.

**Table 2:** Peak visual acuity after fluocinolone acetonide implant segmented by baseline visual acuity and duration of diabetic macular edema (**a**) and peak central retinal thickness after fluocinolone acetonide implant segmented by baseline central retinal thickness (**b**).

**(a)**						
	N * **(Studies)**	**Mean Follow-** **Up (Months)**	**Mean DME ^†^** **Duration (Years)**	**Mean Baseline VA ^‡^ (Letters)**	**Mean Peak** **VA (Letters)**	**Mean Peak VA Gain (Letters)**
Baseline VA (letters)						
<50 letters	14	18.6	3.9	43.7	54.7	11.0
50–60 letters	10	22.4	2.4	54.2	61.4	7.7
>60 letters	5	19.2	2.7	63.4	70.4	7.0
DME duration (years)						
≤2 years	7	24.0	2.0	59.6	68.3	8.7
2–4 years	14	20.9	3.1	47.1	56.4	9.4
>4 years	4	15.0	5.9	53.2	58.3	5.1
**(b)**						
	N * **(Studies)**	**Mean Follow-** **Up (Months)**	**Mean DME ^†^** **Duration (Years)**	**Mean Baseline CRT** **^§^** **(µm)**	**Mean Peak** **CRT (µm)**	**Mean Peak CRT Decrease (%)**
Baseline CRT (µm)						
<400 µm	2	18.0	2.9	349	285	18
400–600 µm	20	22.0	3.3	505	328	34
>600 µm	5	14.0	2.7	640	361	44

* Number; ^†^ Diabetic macular edema; ^‡^ Visual acuity; ^§^ Central Retinal Thickness.

## Data Availability

Data is contained within the article.
